# Against ‘instantaneous’ expertise

**DOI:** 10.1186/s13010-022-00123-3

**Published:** 2022-09-21

**Authors:** Alexander Mebius

**Affiliations:** Division of Philosophy, Royal Institute of Technology (KTH), Teknikringen 76, 114 28 Stockholm, Sweden

**Keywords:** Expertise, Justification, Philosophy of medicine, Biotech, Theranos, Mechanisms

## Abstract

**Background:**

Healthcare is predicated on the use of biotechnology and medical technology, both of which are indispensable in diagnosis, treatment, and most aspects of patient care. It is therefore imperative that justifications for use of new technologies are appropriate, with the technologies working as advertised. In this paper, I consider philosophical accounts of how such justifications are made.

**Methods:**

Critical philosophical reflection and analysis.

**Results:**

I propose that justification in many prominent accounts is based on the designer’s professional experience and on expert testimony. I argue, however, that professional designers are not in a position to justify a new biotechnology or medical device if the justification is based on testimony or past experience of presumably similar technologies. I argue (1) that similarity judgments offered by instantaneous experts cannot be viewed as contributing (epistemically) to evidential justification of new and unproven technologies; and (2) that designers and manufacturers cannot endorse a technology’s effective function in a patient-care context until it has been successfully used in that context.

**Conclusion:**

I show that an expert’s past professional experiences can never predict or justify the impact of a novel technology on human health. This is because any new technology leads to the introduction of new mechanisms with unprecedented functions. The new technology therefore needs to be studied in situ and justified as a newly created mechanism within the relevant healthcare setting. Ultimately, justifications of this type rely on the scientific community and society engaging in repeated experimentation and observation of the technology, and confirming its successful use.

## Background

**Fig. 1:**
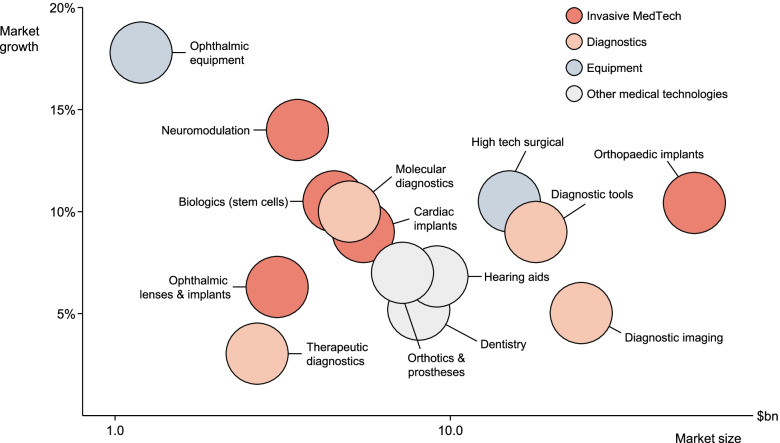
Technologies used in healthcare. Data retrieved from the Orbis Database [1]

Modern healthcare necessitates the use of new technology in most aspects of patient care.^1^ These technologies are deployed in various diagnostic contexts, such as establishing whether a patient is infected with a virus, is pregnant, or is experiencing dangerous side-effects from a treatment. Vaccines and medical implants are among the technologies used for healthcare treatments and procedures. (Figure 1 provides an overview of these various medical technologies and their uses). Furthermore, procedures may themselves require technologies, such as the advanced surgical equipment used to insert a hip implant into a patient’s body. It is therefore vital that technologies work as intended along each step of the treatment pathway.^2^

It is also fair to assume that every technology has an approved function; that the reason for its use has been adequately justified or demonstrated. However, justification can vary according to how and where the technology is applied. For example, taking the same biotechnological tool used to treat certain infectious diseases in farm animals in an agricultural setting and then using it to treat companion animals in a veterinary clinic may not be justified. Similarly, justification for using a technology to diagnose or treat the new disease in humans can differ according to healthcare context.^3^

### Professional designer experience and testimony

The principal source of justification for technological functions and their use cited in the relevant literature is professional expertise and testimony (e.g., [6–8], but c.f [9–11], and [12] for other viewpoints). According to Houkes and Vermaas [6], for example, expert testimony plays a crucial role in justification: ‘Testimony is not just a basis for the effectiveness, capacity and contribution beliefs that are involved in function ascriptions, but also provides a basis for this privileging’ (pp. 113–114).

Houkes and his colleagues also claim that justifications can be made before a technology has been successfully used and/or before the scientific community has accepted it: ‘some artifacts are not bootstrapped through successful use; instead, they jump more or less fully armed from the heads of scientifically informed, professional designers’ ([7], p. 203). Vermaas and Houkes [8] similarly allow for evidential justification of a technology before its successful use in a patient-care context.

They and their colleagues give, as an example, a hypothetical case where use of a vaccine needed to contain an outbreak of disease is ‘instantaneously’ justified on the basis of expert testimony: ‘the Dutch government and hospital staff ... may ground their beliefs about the drug in testimony’ ([7], p. 203). The authors add emphasis to their claim when they state:

‘The important point is that, prior to successful use or even *any* use, the function ascriptions are grounded and the substance can be described as [a] vaccine’ (p. 203).

Some philosophers of medicine have correspondingly pointed out that the professional experience can provide empirical warrant for vindicating analogous treatment decisions:

‘Clinicians often make explicit reference to primary experience, perhaps referencing a particularly memorable or unusual case, or lessons learned from repeated exposures. Specific cases, remembered or recorded, can serve as analogues for a novel case at hand’ ([13], p. 70).

According to this analysis, clinical expertise allows clinicians to make inferences about novel cases from memory of past cases. However, I consider reliance on past expertise or testimony to justify a new technology to be misguided, and I explain why below, along with some examples, in the results and discussion section.

## Methods

This study is an exercise in epistemological philosophy and methodological reasoning. Its method is critical philosophical reflection and analysis. The method is not peculiar to philosophy but common to all rational discussion and therefore the sciences and arts in general. It entails stating one’s problem clearly and examining one’s suggested solutions critically [14].

## Results and discussion

### The case against instantaneous expertise

Arguably, before we can be sure that a technology (let us call it F) does what its designer says it does, there must be justification for that claim. But even then, until the creator of F provides satisfactory evidence as to why we should adopt F, we cannot be confident that F has the function assigned to it. So what provides sufficient reason or evidence for us to accept that F has the designated function?

As noted above, some philosophers of technology claim ‘instantaneous expertise’ provides sufficient reason (justification). According to this analysis, in its basic form, expert designers use their expertise, which is based on their experience of past events, or the experience of another expert (i.e., expert testimony) to justify use of a new technological tool or device. Thus, in accordance with Vermaas and Houkes’ [8] argument, because the professional designers’ respective experiences are what secure justification, they are the people who can make that determination. Vermaas and Houkes also claim, however, that justification can be freely provided by ‘*any* agent who develops and communicates a use plan and who can justify it, if only by plain experience that it works’ ([8], p. 9, emphasis added). Moreover, this allows professional designers to justify prospective technologies prior to their actual successful use in practice.

At this point, it is worth considering Vermaas and Houkes’ [8] analysis of when technological functions need to be justified in more detail:

‘An agent *a* ascribes the capacity to *Φ* as a function to an artefact *x*, relative to a use plan *p* for *x* and relative to an account *A*, iff:

I. the agent *a* has the capacity belief that *x* has the capacity to *Φ*, when manipulated in the execution of *p*, and the agent *a* has the contribution belief that if this execution of *p* leads successfully to its goals, this success is due, in part, to *x*’s capacity to *Φ*;

C. the agent *a* can justify these two beliefs on the basis of *A*; and

E. the agents *d* who developed *p* have intentionally selected *x* for the capacity to *Φ* and have intentionally communicated *p* to other agents *u*’ (p. 9).

The I condition maintains that the agent justifying the technology is confident its functional capacity will actualise when the technology is used according to its use plan. Condition C requires the agent justifying the function to provide evidence for their assertion that the technology will indeed function as intended by the use plan. Condition C therefore requires evidence beyond the agent’s assertion that the technology has a functional capacity (as required by condition I). The additional requirement calls on the agent to use relevant evidence to justify the assertion:

‘The relevant evidence may be experience that the artefact has the capacity, testimony by other agents, or scientific or technological knowledge; in all cases, the evidence supports the function ascription by supporting the beliefs that the artefact has the corresponding physiochemical capacity and that this capacity explains, in part, the effectiveness of a use plan’ ([6], p. 93).

Because the plan is prespecified, the most relevant evidence justifying its use tends to be professional experience and testimony. Other evidence, such as scientific or technological knowledge, can only be evidence collected before the plan is implemented, that is, before the technology is used in practice for the first time. The scientific and technological knowledge may include isolated instances of certain characteristics, such as a material’s ‘physiochemical capacities’, those pertaining to the human body, and the molecular structure of a biotechnology.

However, this type of knowledge does not necessarily translate into empirical support for how these characteristics and the new technology will interact. We can only know this by observing how the interaction plays out in practice. To use a somewhat simplistic analogy, we cannot use our experience of what cheese tastes like and what marmalade tastes like to deduce what the two will taste like when combined, assuming they have not been previously combined (even if an experienced chef might make a sensible conjecture about what the combination could possibly taste like).

In short, we cannot infer the new experience from past experience and especially so if the two turn out to be radically different (e.g., the taste of cheese does not closely approximate the combined taste of cheese and marmalade, and the respective tastes of cheese and marmalade, likewise, are not closely related—because if they were similar enough to be practically indistinguishable it would hardly be possible to say that their combination leads to a qualitatively distinct tasting experience).

To take an actual example from the history of technology, the inventor Alfred Nobel and his colleagues could not have possibly cited their past experience of handling nitroglycerin on the one hand, in conjunction with their past experience of handling diatomaceous earth on the other, to thereby deduce (with no further empirical test or justification being necessary) that the two intermixed turn out to be much safer to handle (than nitroglycerin alone). Nobel’s team may, however, have deduced how this very well could be a possible outcome (a hypothesis), but as such this would still need to be tested.

Generally, then, when we consider evidential justifications (and not merely potentially justifiable posits) of extraordinary technological claims, we often lack relevant reference cases to draw on. This is because extraordinary (prospective) technological claims, unlike ordinary (retrospective) claims, often go well beyond what can be known from past experience and what can be anticipated from that experience. It is still fully possible, however, for an experienced designer or medical expert to recall certain features found in previous instances to formulate a conjecture about a novel case at hand. This would not, however, amount to providing evidence for the proposed existence of a new and effective technological function. Instead, the experts’ past expertise could potentially serve to formulate a hypothesis about how some prospective technology might possibly work.

Accordingly, certain past experiences may generally contribute to the formulation of a hypothesis, but the possible heuristic value of past expertise in proposing what could putatively be achieved should not be confused with any substantive claim to knowledge about the thing being explored. As such, past experience should not be dressed up as factual knowledge about the thing that actually needs to be investigated (or be considered to provide any unique insight regarding unactualised possibilities).^4^

To speak more precisely, if the designer through experience, as it were, already possessed the relevant knowledge, it would hardly be necessary (and perhaps even morally dubious) to conduct further empirical tests. The technology could quite assuredly be introduced with no need for further empirical study. For clearly if one claims to have knowledge that justifies the use of a technology one cannot presume to need to investigate further (viz. Aristotle: ‘for men do not investigate matters about which they know’ [15], NE 6.9, 1140a32–34).

We can doubtless agree that an expert designer can only be an experienced designer, that is, someone who has past experience (memories) of designing, manufacturing, and developing technological products. Because experience is built on the memories of past experiences, a new technology cannot be part of an expert’s experience and therefore that person’s experience cannot be counted as evidence of how the technology will perform. The process of designing biotechnologies or medical devices may, of course, be part of that person’s experience, but experience of the process involved in developing a technology and experience of how it functions once produced are very different.^5^

**Fig. 2:**
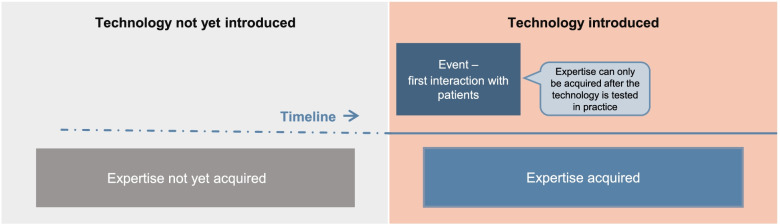
Acquisition of expertise

We can describe being an expert on F as being in a state of knowing F. Knowing that the technology has a function is a state; thus, the state of knowing F, of knowing that the technology has a function. However, if I am learning about F, then I have not yet learned F: if I am building a house, I have not yet built it (see Fig. 2). I may quickly learn about the technology (that it performs function F), but I cannot be an expert on F while I am still learning about it (again, I cannot have built the house if I am still building it). I cannot at the same time be in a state of becoming an expert on F while being an expert on F. Also, while learning about F, I may be interrupted. I am therefore not an expert on F until I have reached a terminus. In this respect, there is no instantaneous acquisition of expertise. Becoming an expert takes time; being an expert does not.

Note that I am not referring here to the problem of inferring from past experience to future events (or the related problem of making an inference from a single occurrence to a general statement). Rather, my discussion concerns the problem of making an inference about a future event despite having *no* past experience of that event. Likewise, we cannot use testimony about part of a mechanism that has not existed in the past to make inferences about how the new mechanism will perform in the future. The new mechanism is not only new technology but new technology in a new setting. By changing or disrupting an existing mechanism consisting of specific elements, the new technology changes how those elements are organised and operate. The new mechanism will thus serve a new effective function.

### Examples of unjustified technological use in patient care

As previously noted, acquiring expertise and becoming an expert is a process that takes actual experience. The absence of actual experience of successful technological use prior to a technology’s introduction is illustrated in the following examples, in which instantaneous expertise is primarily relied upon for evidential justification.

In 2013, engineers at the Silicon Valley medical technology company Theranos announced they were ready to use their revolutionary diagnostic device on patients for the first time. The company, led by Elizabeth Holmes, had an agreement with Walgreens (a chain of pharmacy stores) to make the company’s technology available to Walgreens’ customers. Holmes claimed that the new technology, ‘the MiniLab’, which she also analogously referred to as ‘the iPod of Healthcare’, would enable fast and accurate results from just a drop or two of blood pricked from the finger. She also claimed that Theranos’s new technology would perform the unprecedented operation of allowing multiple tests to be analysed simultaneously from just a single sample [16].

The company had patented and developed the technology for more than a decade before its launch. Leading experts had vouched for the company’s technology and well-known public figures such as Henry Kissinger had endorsed the new purported product’s revolutionary potential for society (incidentally, the former U.S. Secretary of State Henry Kissinger was a member of the company’s board). In 2014, Theranos was valued at US$9 billion.

However, by 2015, the scientific community were raising concerns about the evidential justification assigned to the functional accuracy and reliability of Theranos’s blood-testing device [17]. In 2016, a study published in the *Journal of Clinical Investigation* indicated that Theranos’s blood test results were ‘outside their normal range 1.6× more often than other testing services ... [and] showed significant interservice variability’ ([18], p. 1734). The study results put into question whether the device had the actual effective function the Theranos designers had assigned to it and justified in accordance with their expertise. In September 2018, the company was liquidated after several reports found Theranos’s technology did not actually perform at all in the way it was thought it would perform.^6^

To take another well-known example, consider vertebroplasty, a procedure still used for alleviating pain caused by a bone fracture. The procedure consists of injecting the bone cement polymethyl methacrylate into the patient’s fractured bone. Vertebroplasty was introduced during the mid-1990s after numerous experts reported remarkable success with this technology (e.g., [19, 20]). Expert testimony led Medicare to reimburse the procedure in 2001 [21]. By 2004, the number of vertebroplasties being performed in the United States alone were 24,000. In 2009, however, the scientific community performed two clinical trials designed to establish whether the technology was working as well as countless expert testimonies and ‘hands-on’ experience had suggested. The results of these two independent experiments, which were published in the *New England Journal of Medicine*, showed that the technology worked no better than a placebo (i.e., the sham procedure used with a control group of patients) [22, 23].

## Conclusions

In this article, I endeavoured to show that drawing on professional expertise as justification for new technologies used in healthcare settings is misguided. It was argued that evidential justification of instantaneous expertise itself is based on a presumed analogy with past experience which needs to be justified. This conclusion may also apply to justification for using innovative technologies in other areas that have an impact on human health, such as, transportation, sanitation and agriculture.^7^ Ultimately, the introduction of a prospective technology is an extraordinary or unparalleled event, and how well the technology works as anticipated cannot be experienced by experts until the first actual patient interaction with that technology takes place. Only then can the community of scientific or technological experts possibly recognise the new technology as having the justified function. The evidence need not be conclusive, but it must be justified empirical evidence based on actual successful technological use.

## Data Availability

Not applicable.

## Footnotes

1 In this paper I use the terms ‘biotechnology’ and/or ‘medical technology’ to refer to any technologies that are used for medical purposes in various healthcare contexts and settings, including hospitals and clinics, patients’ own homes, care homes, dental clinics, and so on. The discussion thus relates to the technologies, whether health, medical, or other, used during patient care.

2 The combined estimated revenue of the global biotechnology and medical technology industries exceeded US$200 billion in 2019 (estimate based on combined revenue figures of 1440 biotechnology and medical technology companies in 2019; data retrieved from Orbis database [1]). It is therefore of interest to consumers, governments and/or taxpayers that these funds are appropriately allocated.

3 An experiment will often be epistemically preferable to non-experimental observation for action guiding (i.e., technological) purposes [2–4]; see also, [5], for a related discussion on the role of medical expertise in action-guiding experiments).

4 As an anonymous reviewer correspondingly noted: ‘Having sat on scientific review panels for clinical trials, it has always surprised me that some treatments (e.g., autologous stem cell injections to hasten healing of joint repair procedures) become standard of care without ever having gone through comparative clinical trials’.

5 In the worst-case situation, an appeal to an expert can become a tautology or close to one: X says that F because X says that is the case (X knows F because X knows F). However, this fallacy can be avoided if the appeal is made not to the expert but to his or her expertise. X has experienced F in the past and provides a testimony of this experience. His or her experience of F having worked forms the basis of an expert testimony. The testimony that F has worked can be used as evidence that F has worked on at least one occasion in the past.

6 To be sure, strong justification from repeated tests is ultimately difficult to ignore: ‘You may have all kinds of reasons for believing that a drug helps against a particular disease, for instance biochemical reasons, belief in someone’s authority, or belief in the supernatural powers of the plant that the drug was made from. But if a competently performed experiment shows that the drug does not have the effect in question, then that is a result you cannot reasonably dismiss’ ([3], p. 619).

7 See, for instance, Hansson [24] on this topic for an engaging discussion of the risks involved in justifying promising but unproven molecular biotechnologies such as precision editing of nucleotides within existing genes.
